# Duodenitis

**Published:** 1875-11

**Authors:** A. L. Knight

**Affiliations:** West Columbia, West Virginia


VOL. V.—NOVEMBER, 1875.—NO. 11.

### Original Communications.

### DUODENITIS.

*Read before the Meigs and Mason Academy of Medicine, Au[illegible] 12, 1875, and Requested to be Published.*

*Mr. President and Gentlemen:*

At the request of a member of this institution, I offer you a short paper upon acute inflammation of the duodenum—a disease so rare that a majority of our text-books are either silent upon it, or treat it under the caption of gasto-enteritis.

Professor Wood has given a very good description of duodenitis in his practice. (Vol. 1, p. 557. Edition of 1847.) So far as the essential symptoms in perhaps the greatest number of cases met with, but to my mind stops short of some of the gravest complications often attending.

I will give his diagnostic symptoms in full as a basis of this discussion :

"Duodenitis, when it occurs, is almost always associated with inflammation of the stomach. Its existence may be suspected when, in addition to the ordinary symptoms of gastritis, there is deep-seated pain in the vicinity of the pylorus extending below the stomach, towards the left hypochondrium, or pain in the back about the first or second lumbar vertebra; when there is tenderness upon strong pressure in the space, which lies immediately to the left of the right hypochondrium, without any evidence of enlarged or inflamed liver, and when the skin is more or less yellow and the urine bilious as in jaundice. If these symptoms should occur without vomiting, great thirst or pain, and tenderness in the region of the stomach, indicating gastritis, without diarrhœa, pain in the lower bowels, or tympanitis, indicating enteritis, and without enlargement or tenderness of the liver, indicating hepatitis, it may be inferred the inflammation is confined to the duodenum.

"In acute duodenitis, there is occasionally along with the fever a degree of coma dependent probably upon the hepatic derangement; in the chronic form of the complaint, a diagnostic symptom is the occurrence of pain two or three hours after a meal, arising from the passage of a portion of the contents of the stomach into the duodenum.

"The bowels in duodenitis are generally slow, but readily acted on by cathartics.

"The appearances after death are so similar to those in gastritis as not to require particular notice except in relation to the mucous follicles, or as they are often called, glands of Brünner, which are very numerous in the duodenum, especially near the pylorus, and in inflammation of this viscus are apt to be enlarged, exhibiting sometimes elevated irregular, almost continuous patches of considerable extent.

"The causes of duodenitis are essentially the same as those of inflammation of the stomach; such of them, however, as act through the liver are probably more influential in producing the former than the latter affection."

The foregoing is certainly a very summary manner of diagnosing and discussing an inflammation, having its seat in a viscus so intimately connected with the functional products of an organ so essential in modifying the chyme for lymphatic metamorphosis and healthy assimilation or circulation.

It will be noticed that the argument does not claim entire biliary obstruction and suppression of bile in producing the icterus condition of the skin, from which we infer a want of a complete elimination of that element in the functional action of the biliary organ, owing, perhaps, to an altered condition of the blood pabulum through the primary malaction of the glands of Brünner, called by some the minor pancreas, and also to defective secretion and admixture of the stimulating and anticeptic properties of healthy bile.

That this disordered action of the liver precedes the inflammation of the duodenum from other causes, as spoken of by Professor Wood, just quoted, I am not prepared to deny, but should think, were the proposition true, that the disease in question would be of much more frequent occurrence than it is, as that organ, as are all glandular structures easily deranged by improper diet, exercise and vicissitudes of temperature.

We should regard it as more probable that a catarrhal idiosyncrasy predisposing to inflammations of the mucous and submucous membranes might occasionally take an inflammation centered in the short viscus called the duodenum, giving rise to the symptoms just quoted, as well as those which we propose to add before closing this paper, and (perhaps it makes no essential difference in what manner the bile is suppressed or obstructed, if obstructed in the complaint, or whether it be suppressed or obstructed at all, provided, there be certain other symptoms indicating this particular inflammation, including those already enumerated, which, however, should be differentiated from inspisated cholesterine or gall stones, where they irritate the biliary ducts in a tedious passage into the duodenum, giving rise to considerable irritative fever, as in several cases falling under my own observation, accompanied with slight mental disturbance in which there could be no mistake, after the fact, as the concretions were subsequently passed with the fæces, the general symptoms being those of duodenitis, except the pains were more in the right hypochondrium, spasmodic and intermitting. These, it is true, are but slight differential signs, yet when contrasted with the more pointed symptoms of acute duodenitis are quite sufficient. Other forms of jaundice, unaccompanied with continued irritative fever, or if so, but slightly, with little or no cerebral disturbance, are not likely to be confounded with the disease in question, except those attended with hemorrhage, so excellently described by Professor Watson, (3 Am. edition, p. 769,) under hæmatemesis, more particular malæna, the difference is that the blood, or blood-like, is in duodenitis always discharged per anum and not per orem, as is sometimes the case in malæna.

My principal notions of acute duodenitis are derived from observations of four cases in my own practice, and the report of a like number from the observation of others, one of which was described to me by Mr. D. W. Dabney, formally a student of mine, seen by him in a clinic (if my memory serves me,) in the Miami Medical College, at Cincinnati, which has no further interest than presenting some symptoms not enumerated by Professor Wood, and which were also noted in two of the three other cases reported. I regret that I cannot now refer you to the authority from which I learned the facts of these extended symptoms with the pathological conditions shown up in the two cases just mentioned by post-mortem examinations, and I am sorry that two of my own cases passed away without their pathology being fortified by observations upon the cadaver.

The symptoms alluded to were hemorrhages and melænic discharges from the bowels, gradually changing in the course of the disease into a peculiar lead colored, thin and fœtid dejections, often attended with hiccoughs. It should have been remarked, that in those cases, at the onset of the inflammation, the liver had a doughy feel without tenderness upon strong pressure. The pulse in the beginning of acute duodenitis is pretty uniformly like that in gastritis, small and feeble, but rarely so much quickened, often not exceeding ninety per minute. Where both these visceres are inflamed, it is sometimes almost imperceptible, and apt to betray us into improper treatment, as will be shown before closing this paper.

In the post-mortems referred to, in which there had been hemorrhages and the peculiar stools alluded to, there were found, in addition to the irregular elevated patches of Brünner's glands, ulcerations of the same confined in one case to the duodenum, and in the other, involved those about and within the pyloric orifice of the stomach, with thickening of the mucous coat of the duodenum.

These cases, like the two fatal ones in my own practice, lingered from two to three weeks—a much longer time than usually elapses in gastritis or in gastro-duodenitis.

That which lends the greatest interest to duodenitis, having all or most of the above symptoms, and continuing over ten or twelve days, with but slight icterus, is its great similarity in appearance to some of the lower forms of malarial or hemorrhage miasmatic fevers, as well as some phases of typhoid, enteric fever. Mistaking it for either, and more especially for the latter, would certainly be very prejudicial to its successful treatment. Fortunately, this mistake would seldom occur if a careful examination is made of the lower bowels in the onset of the disease, before tympanitis supervenes from the decomposition of the sanious excretions of the upper bowel, evolving gas sufficient to distend the abdomen, and even then the mistake would be somewhat inexcusable, for the pain would not be so increased by pressure as it would be in typhoid fever, add to which the non-prevalence of the latter disease at that given time.

"The treatment in acute duodenitis," says Wood, "is much like that for gastritis." If we followed the practice of twenty years ago, (let me ask is it the best that could be adopted?) it would consist of blood-letting general and local, saline cathartics, cold sponging, with mercurial alteratives in the early stages of the disease, to which there should soon be added blisters to the hypochondrium, and continued until tonics and gentle stimulants would be indicated. How many of the present day would have the confidence and boldness to strictly follow such examples? Why not? We are told not to fear the consequences, that the heart's action will improve under it, and if we stand idly by, or treat it on the expectant plan, our worst fears will be realized.

In my own practice the above treatment has been closely followed, with results as before stated. In the two cases which recovered, after the first few days, a gentle saline cathartic every other day, with alterant doses of calomel, were given, alternating with tonics and mereral astringents as they seemed indicated. I have been partial to the tincture guaic, rendered more bland by the addition of syrup rhei or acacia, with ammonia carbonate, quantity regulated *pro re nata*.

I would not now recommend general bleeding in this disease, since experience proves that venesection does not proportionately control mucous inflammation as it does in inflammations of the serous tissues. Local bleeding and early blistering I should regard as preferable.

Had I a case now in charge, I should rely upon alternating doses of mercury, minute but oft repeated salines; the latter for their solvent and antiphlogistic powers, as well as their local action upon the affected bowel, and should not hesitate to push them to purgation once or twice weekly, calling to my aid stimulants and tonics, especially those stimulants referred to, as they become indicated.

The vital powers in duodenitis often seem rapidly to ebb away, and sometimes quite unexpectedly; drastic cathartic, or even calomel, in full doses, after the first few days of the disease, is generally followed by syncope and death. It so happened in one of my fatal cases, after passing from my care to that of a neighboring practitioner, who took charge of the case and insisted that I was incorrect in my diagnosis.

It may be thought that I have not been sufficiently clear in my differential symptoms of this disease and that of melæna. Suffice it to say, that melæna is not usually attended with a high grade of fever, being generally, if not always, dependent upon viceral congestions, especially those connected with the portal veins, but disconnected with congestion of the hepatic veins; and further, should inflammation succeed melæna, followed by the icterus hue, (I mean icterus, not icteroid hue, for the yellowness in duodenitis has a peculiar bronze-like cast.) I should regard it as duodenitis, having its origin in congestion and irritation of that locality, even were the hepatic veins unimplicated.

Taken in that light, the disease is not so rare as we have been led to suppose.

Hoping my paper will at least command your thoughtful attention, and that you will investigate any case or cases having those symptoms, and report your observations to this academy,

I am respectfully yours,

A. L. KNIGHT, M. D.

*West Columbia, West Virginia.*

